# The Iowa State Dental Society

**Published:** 1865-09

**Authors:** 


### THE IOWA STATE DENTAL SOCIETY.

Held its regular annual meeting in Dubuque, Iowa, on the 19th of July last.

A larger number was present than ever before. This society is one of the most active, and, though young, the amount and character of work accomplished at this meeting, we think, has hardly been excelled by any similar society. The report of the discussions, a portion of which we give in this number, together with the essays read, show something of the work done and the interest manifested.

Everything was laid out on a broad scale, and taken hold of with a vigorous hand. This society promises soon to be one of the most prosperous and efficient in the country.

We know of no society that has matured so rapidly as this, and still we feel certain that its growth is permanent.

There are some very active members whose aspirations will not be satisfied till it is made capable of accomplishing the greatest good within the range of its operations.

